# Disease-specific alteration of karyopherin-α subtype establishes feed-forward oncogenic signaling in head and neck squamous cell carcinoma

**DOI:** 10.1038/s41388-019-1137-3

**Published:** 2019-12-10

**Authors:** Masaharu Hazawa, Kie Sakai, Akiko Kobayashi, Hironori Yoshino, Yoshihiro Iga, Yuki Iwashima, Kee Sing Lim, Dominic Chih-Cheng Voon, Yan-Yi Jiang, Shin-ichi Horike, De-Chen Lin, Richard W. Wong

**Affiliations:** 1 Cell-Bionomics Research Unit, Institute for Frontier Science Initiative, Kanazawa University, Kanazawa, Ishikawa Japan; 2 Laboratory of Molecular Cell Biology, School of Natural System, Institute of Science and Engineering, Kanazawa University, Kanazawa, Ishikawa Japan; 3 WPI Nano Life Science Institute, Kanazawa University, Kanazawa, Ishikawa Japan; 4 Department of Radiation Science, Hirosaki University Graduate School of Health Sciences, 66-1 Hon-cho, Hirosaki, Aomori 036-8564 Japan; 5 Cancer Model Research Innovative Unit, Institute for Frontier Science Initiative, Kanazawa University, Kanazawa, Japan; 6 Department of Medicine, Cedars-Sinai Medical Center, Los Angeles, CA 90048 USA; 7 Advanced Science Research Center, Institute for Gene Research, Kanazawa University, Kanazawa, Ishikawa Japan

**Keywords:** Cancer genetics, Protein transport

## Abstract

Nuclear import, mediated in part by karyopherin-α (KPNA)/importin-α subtypes, regulates transcription factor access to the genome and determines cell fate. However, the cancer-specific changes of KPNA subtypes and the relevancy in cancer biology remain largely unknown. Here, we report that *KPNA4*, encoding karyopherin-α4 (KPNA4), is exclusively amplified and overexpressed in head and neck of squamous cell carcinoma (HNSCC). Depletion of KPNA4 attenuated nuclear localization signal-dependent transport activity and suppressed malignant phenotypes and induced epidermal differentiation. Mechanistically, KPNA4-mediated nuclear transport of Ras-responsive element-binding protein (RREB1), which sustains Ras/ERK pathway signaling through repressing miR-143/145 expression. Notably, MAPK signaling enhanced trafficking activity of KPNA4 via phosphorylation of KPNA4 at Ser60. These data reveal that KPNA4 establishes a feed-forward cascade that potentiates Ras/ERK signaling in HNSCC.

## Introduction

Head and neck squamous cell carcinoma (HNSCC) is lethal malignancies arising from oral squamous epithelium. HNSCC is characterized by aggressive behavior such as metastasis and recurrence. Previous studies highlighted a key subset of oncogenic transcription factors (TFs) and gene targets in human SCCs including HNSCC [1–7]. Current gene-expression network approaches commonly focus on TFs, biasing network-based discovery efforts away from potentially important non-TF proteins.

The nuclear pore complex (NPC) is constructed by 30 nucleoporin (NUP) proteins and establishes the sole gateway between the nucleus and the cytoplasm. Systematic nucleocytoplasmic transport occurs through NPCs on the nuclear envelope [8–11]. Accumulating evidence has suggested that tissue/disease-specific NUP arrangements determine cell fate [12–18]. We recently reported that expression levels of NUP62, which regulates nuclear transport of the oncogenic TF p63, are elevated in SCCs [16]. Later, Roriguez-Bravo et al. further showed that overexpressed POM121, a transmembrane NPC component, drives nuclear import of oncogenic TFs and promotes lethal prostate cancer [17]. Together these findings suggest that cancer-specific alterations of nuclear transport factors may facilitate the transduction of oncogenic signaling.

Nuclear transport receptors (NTRs), such as karyopherin-α (KPNA) and karyopherin-β (KPNB) (also known as importins) family members, selectively aid the shuttle of karyophilic cargo proteins, which harbor nuclear localization signals (NLSs), through NPCs [19–21]. The human KPNA family consists of 7 subtypes and each KPNA has cargo specificity [21]. Although the significance of KPNA subtypes during differentiation and development has been established [22, 23]. Onset NTRs abnormality of human HNSCC and its significance remain unknown.

Here, we performed genomic profiling of KPNA family members in HNSCC and identified *KPNA4*, encoding karyopherin-α4 (KPNA4), as a specifically amplified and overexpressed KPNA subtype in HNSCC. KPNA4 is required for prevention of epidermal differentiation and activation of the Ras/MAPK pathway through mediating nuclear transport of Ras-responsive element-binding protein (RREB1). Further, MAPK-dependent phosphorylation of importin-β binding (IBB) domain of KPNA4 enhances its traffic activity, resulting in feed-forward loop of MAPK pathway in HNSCC.

## Results

### Identification of *KPNA4* as a HNSCC-specifically altered gene

**Fig. 1:**
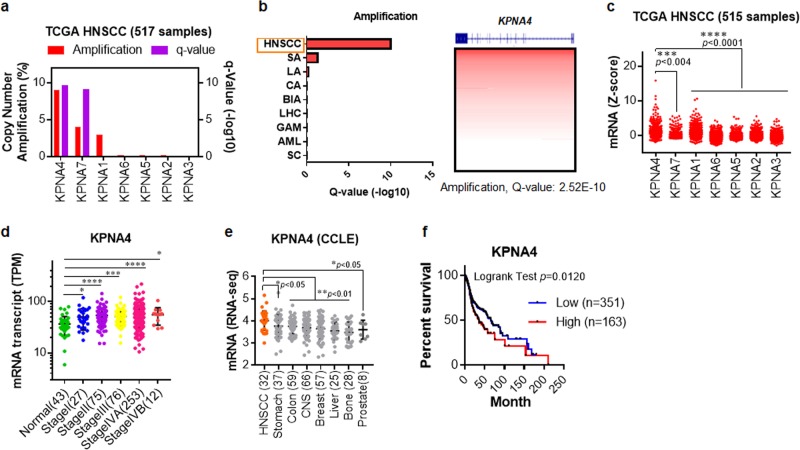
Profiling of KPNA alteration in HNSCC. **a** Analysis of *KPNA* copy number alteration (CNA) in HNSCC from TCGA (http://cancergenome.nih.gov/). **b** Summary of *KPNA4* amplification across different tumor types from TCGA. SA stomach adenocarcinoma, LA lung adenocarcinoma, CA colon adenocarcinoma, BIA breast invasive adenocarcinoma, LHC liver hepatocellular carcinoma, GM glioblastoma multiform, AML acute myeloid leukemia, SC sarcoma. **c** The expression of KPNA family transcripts in HNSCC from TCGA. **d** The expression of KPNA4 in nontumor tissue, and HNSCC samples from Cancer RNA-Seq Nexus (http://syslab4.nchu.edu.tw/). SI, SII, SIII, SIV denote stages I, II, III and IV. **e** KPNA4 mRNA expression across different types of cancer cells from CCLE (http://portals.broadinstitute.org/ccle/). **f** High KPNA4 expression (mRNA expression *z*-Scores (RNA-Seq V2 RSEM) >mean +2.0SD) was associated with poor disease-free survival of HNSCC patients in the TCGA cohorts.

To comprehensively profile the genetic abnormalities affecting *KPNA*, the Cancer Genome Atlas (TCGA) was re analyzed. The results showed that *KPNA4* was the most amplified in HNSCC (Fig. 1a). Analysis of KPNA4 amplification across multiple cancers revealed that HNSCC showed remarkable amplification (Fig. 1b), which was also observed other types of SCCs arising from lung and cervix (data not shown). We next analyzed TCGA RNA-seq data to examine mRNA levels of each KPNA subtype. The mRNA expression level of KPNA4 was markedly higher than other KPNA subtypes (Fig. 1c). Next, we compared the KPNA4 transcript amounts between 433 cases of HNSCC patients with various pathological stages and 43 cases of normal subjects via Cancer RNA-Seq Nexus (See “URLs”). The analysis revealed that KPNA4 is significantly elevated in HNSCC patients (Fig. 1d). Notably, KPNA4 mRNA levels were especially higher in HNSCC based on Cancer Cell Line Encyclopedia datasets (Fig. 1e). Kaplan–Meier analysis on the TCGA cohorts further revealed that the upregulation of KPNA4 was significantly correlated with poorer outcome of HNSCC patients (Fig. 1f). Collectively, these findings suggested that, within KPNA family, KPNA4 is uniquely upregulated in HNSCC.

### KPNA4 is required for cellular malignancy and regulates global gene-expression network of HNSCC cells

**Fig. 2:**
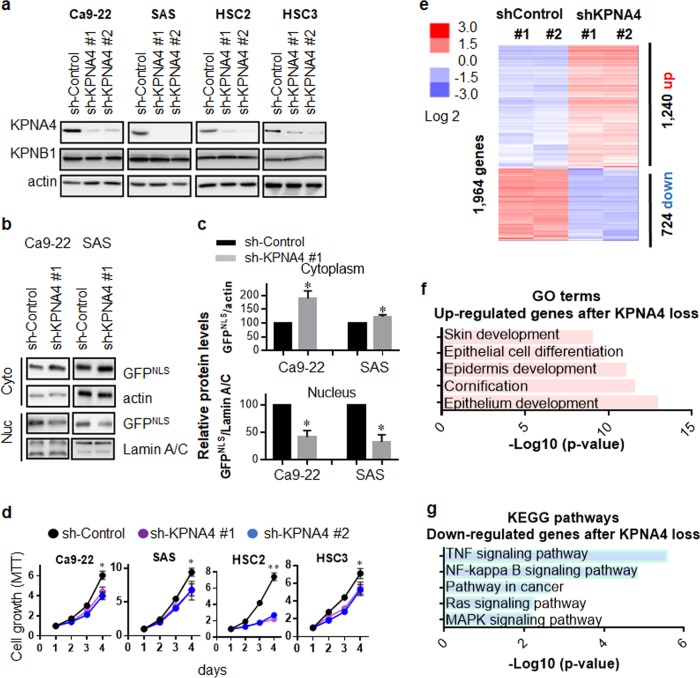
KPNA4 mediates cellular malignant phenotype and affects gene expression in HNSCC. **a** Western blot analysis of each KPNA4 as well as KPNB1 in various HNSCC cell lines expressing shRNA for KPNA4. **b** Western blot analysis of GFP^NLS^ in both cytoplasmic and nucleus fraction. **c** Quantification of the protein levels were performed. Data show mean ± SD from three independent experiments (*n* = 3). One sample *t* test was performed using GraphPad QuickCalcs. *p < 0.05. **d** Short-term proliferation was analyzed MTT assay Data represent means ± SD (*n* = 3). **e** The heat map from microarray data showing upregulated and downregulated genes after knockdown of KPNA4. **f** GO analysis of the upregulated genes upon KPNA4 depletion. **g** Pathway analysis of the downregulated genes after KPNA4 knockdown.

To study the biological role of KPNA4 in HNSCC, we silenced KPNA4 in several HNSCC cell lines by shRNA-mediated knockdown (Fig. 2a). First, we investigated NLS-dependent nuclear transport in those cells since NLS-dependent nuclear import is dominantly regulated by Karyopheryin family [24]. To investigate the role of KPNA4 on nuclear import ability, GFP harboring SV40-derived NLS (GFP^NLS^) was generated (Supplementary Fig. 1) and expressed to HNSCC cells. We found that KPNA4 silencing caused reduction of nuclear GFP^NLS^ amounts while cytoplasmic GFP^NLS^ levels was increased upon KPNA4 knockdown (Fig. 2b, c), suggesting KPNA4 mediates NLS-dependent nuclear imports [24].

Next, we investigated whether KPNA4 is functionally involved in cancer cell biology. Silencing of KPNA4 resulted in a significant reduction in short-term cell proliferation, foci formation, migration ability and resistance to radiation in HNSCC cells (Fig. 2d and Supplementary Fig. 2). Importazol, an inhibitor of KPNB1 [25], also inhibited cell proliferation (Supplementary Fig. 3a). Further, siRNA-mediated KPNB1 silencing resulted in decreased cellular growth (Supplementary Fig. 3b, c), suggesting that KPNA4- and KPNB1-mediated nuclear traffic is required for HNSCC proliferation.

To explore the biological processes and signaling pathways regulated by KPNA4, we next performed cDNA microarray in Ca9-22 cells silenced for KPNA4 (Fig. 2e). Gene ontology (GO) analysis revealed that prevention of KPNA4-dependent transport increased a subset of genes highly enriched in processes regulating epidermal development and differentiation (Fig. 2f). Pathway enrichment analysis indicated NF-κB signaling, Ras signaling and MAPK signaling were affected by silencing of KPNA4 (Fig. 2g).

### KPNA4 prevents epidermal differentiation in HNSCC cells

**Fig. 3:**
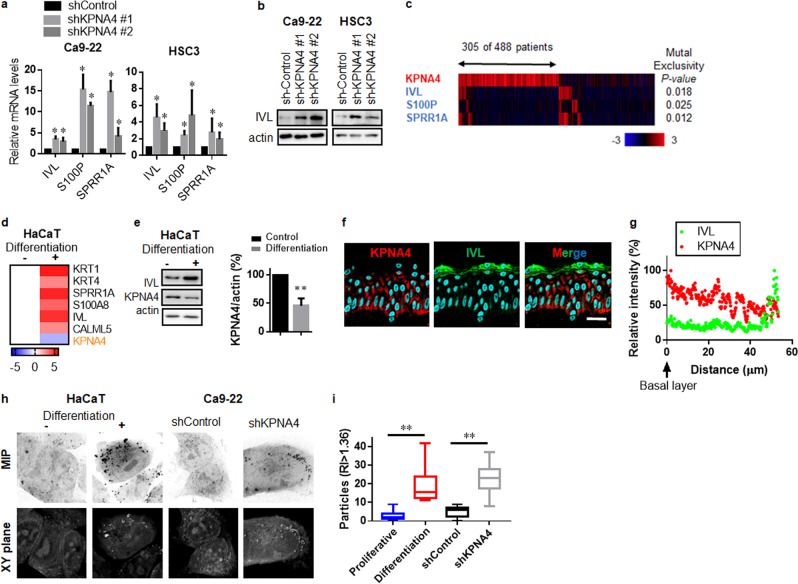
KPNA4 prevent epidermal differentiation in HNSCCs. Epidermal-differentiation genes were analyzed by qRT-PCR (**a**) and western blot analysis (**b**) in HNSCCs cells. qRT-PCR data represent means ± SD (*n* = 3). **c** The heat map showing mutual exclusivity between KPNA4 expression and differentiation-related genes. Samples were divided according to mRNA expression levels [mRNA expression z-Scores (RNA-Seq V2 RSEM) >mean +2.0SD] from the TCGA cohorts. *P* values are based on the fisher exact test. Epidermal-differentiation genes and KPNA4 were analyzed by qRT-PCR (**d**) and western blot analysis (**e**, left) and quantification of KPNA4 proteins levels (**e**, right) in HaCaT cells. Data represent means (qRT-PCR, *n* = 3) or means ± SD (Western Blot, *n* = 3). **f**, **g** Expression profiles of KPNA4 and IVL in normal skin tissue (**f**). Bar = 30 μm. Fluorescent intensity was quantified (**g**). **h**, **i** Three-dimensional reflective index tomographic analysis (**h**) of HaCaT and HNSCC cells. MIP means maximum intensity projection. **i** Quantif**i**cation of granulated particles were analyzed using TomoStudio. The two-tailed Student’s *t* test was used to analyze the potential statistical difference between two groups. *p < 0.05.

GO analysis indicated that epidermal differentiation was the most significantly enriched process activated by depletion of KPNA4 in HNSCC cells. Therefore, we next evaluated the role of KPNA4 in cell fate regulation in HNSCC cells. We found that silencing KPNA4 induced the protein levels of components of the epidermal differentiation complex, including involucrin, calcium-binding protein S100P and small proline-rich proteins 1A (SPRR1A) in HNSCCs (Fig. 3a, b). Examination of TCGA RNA-seq datasets further showed that expression levels of these differentiation genes are inversely related to levels of KPNA4 (Fig. 3c), suggesting that KPNA4 suppresses epidermal differentiation gene expression.

To determine the expression profile of KPNA4 during epidermal differentiation, we next analyzed KPNA4 expression levels in HaCaT cells (nonmalignant keratinocytes) upon in vitro differentiation stimuli. We found that expression levels of KPNA4 decreased while epidermal differentiation genes increased after in vitro differentiation in HaCaT cells (Fig. 3d, e). We also performed immunofluorescent confocal microscopic analysis using healthy human skin samples and found that KPNA4 was expressed in the undifferentiated layer (Fig. 3f, g). Furthermore, three-dimensional reflective index tomographic analysis found granulated contents with a high reflective index (>1.6), which presents in the granule layer of skin, in both differentiated HaCaT cells and KPNA4-silenced HNSCC cells (Fig. 3h, i). Collectively, these results suggest that KPNA4 functions to prevent differentiation in HNSCCs.

### KPNA4 regulates RREB1 nuclear transport to establish feed-forward MAPK signaling in HNSCCs

**Fig. 4:**
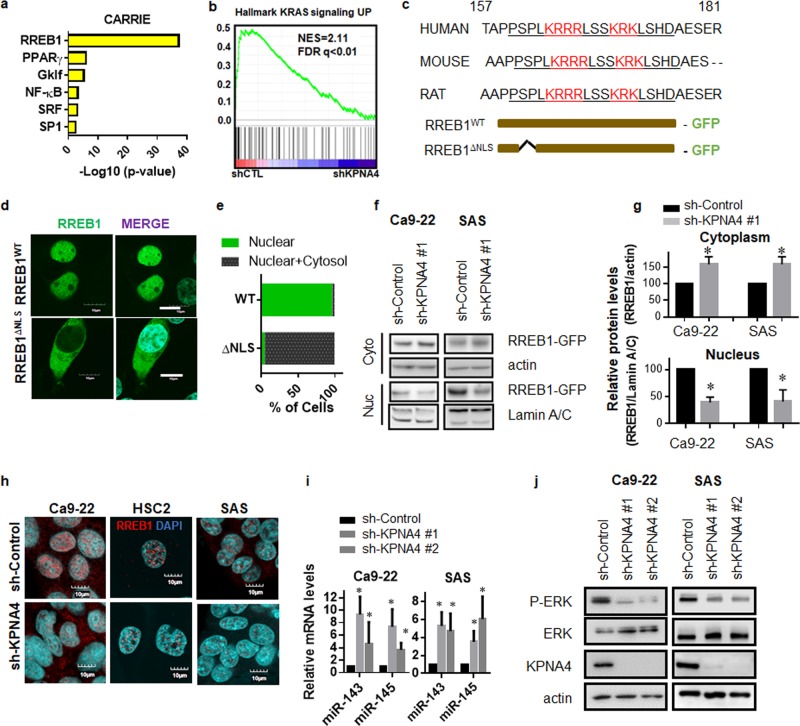
KPNA4 mediates RREB1 nuclear transport to maintain MAPK signaling through suppressing miRNA143/145 in HNSCCs. **a** CARRIE analysis based on gene-expression profile in Ca9-22 cells expressing shRNA for KPNA4 (see “URLs”). **b** GSEA of KPNA4 knockdown microarray datasets (see “URLs”). **c** Predicted NLS in KPNA4 by NLS mapper (upper panel). Diagram of mutation conducted in RREB1 (bottom panel). **d**, **e** HEK293T cells were transfected either RREB1^WT^ or RREB1^ΔNLS^. Fluorescence confocal microscopic analysis of RREB1^WT^ and RREB1^ΔNLS^ [Bar = 10 μm] were examined by confocal microscopy (**d**), and phenotypes are quantified by counting (**e**). **f** Western blot analysis of RREB-GFP in both cytoplasmic and nucleus fraction. **g** Quantification of the protein levels were performed. Data show mean ± SD from three independent experiments (*n* = 3). One sample *t* test was performed using GraphPad QuickCalcs. *p < 0.05, **p < 0.01. **h** Fluorescence confocal microscopic analysis of endogenous RREB1 [Bar = 10 μm] was examined by confocal microscopy. **i** qRT-PCR analysis of m**i**R-143 and miR-145 in HNSCC cell lines depleted of KPNA4. Expression levels of cells expressing nontargeting shRNA is considered 100%. Data show mean ± SD from three independent experiments (*n* = 3). **j** Western blot a*n*alysis of phosphorylated form of ERK1/2 in HNSCC expressing shRNA for KPNA4. Representative image from three independent experiments was shown.

To identify the transcriptional regulatory network established by KPNA4, Computational Ascertainment of Regulatory Relationships Inferred from Expression (CARRIE) was performed based on our cDNA microarray results in Ca9-22 cells (Fig. 4a) [26]. This approach identified Ras-responsive element-binding protein (RREB1) as the most potent cargo regulated by KPNA4 (Fig. 4a). In addition, both gene set enrichment analysis (GSEA) and pathway analysis highlighted Ras/MAPK pathway as significantly affected upon KPNA4 knockdown (Figs. 2g and 4b). Previous studies demonstrated that RREB1 establishes oncogenic Ras/MAPK signaling through repressing miR-143/145 expression [27, 28]. However, the mechanism of RREB1 nuclear transport remains unknown. Therefore, we next investigated whether KPNA4 regulates RREB1 nuclear transport and impacts Ras/MAPK signaling. We first searched for a NLS in RREB1 using the NLS mapper (http://nls-mapper.iab.keio.ac.jp) and found a potential well-conserved NLS in the N-terminus of RREB1 (Fig. 4c). We generated constructs expressing either full-length RREB1 (RREB1^WT^) or NLS-deleted RREB1 (RREB1^ΔNLS^) fused with GFP at the C-terminus (Fig. 4c). Fluorescent confocal microscopy analysis showed that the vast majority of RREB1^WT^ localized in the nucleus (Fig. 4d). In contrast, ~90% cells expressing RREB1^ΔNLS^ showed cytoplasmic fluorescence, indicating RREB1^ΔNLS^ was defective in nuclear localization (Fig. 4d, e). Next we analyzed nuclear import of GFP-tagged RREB1 in HNSCC cells expressing shRNA targeting KPNA4 (Fig. 4f, g). quantification by immunoblotting showed that nuclear amounts of GFP-RREB decreased while cytoplasmic levels of GFP-RREB1 increased by KNA4 depletion (Fig. 4f, g). Furthermore, concordant with our transcriptome analysis, we found that nuclear RREB1 levels greatly decreased after KPNA4 depletion (Fig. 4h), suggesting that KPNA4 mediates RREB1 nuclear transport. We also found that KPNA4-depleted cells showed increased amounts of miR-143/145 (Fig. 4i) as well as reduction of MAPK activity (Fig. 4j), both of which are targets of RREB1 [27, 28]. Consistent with previous reports, silencing RREB1 using siRNA enhanced miR-143/145 expression levels and blocked MAPK activity, and we also observed a suppressed proliferation of HNSCC cells (Supplementary Fig. 4). Taken together, these data suggest that KPNA4 establishes Ras/MAPK signaling through RREB1-mediated repression of miR143/145 in HNSCCs.

### Ras/ERK pathway mediates KPNA4 activity through phosphorylation of S60 in KPNA4

**Fig. 5:**
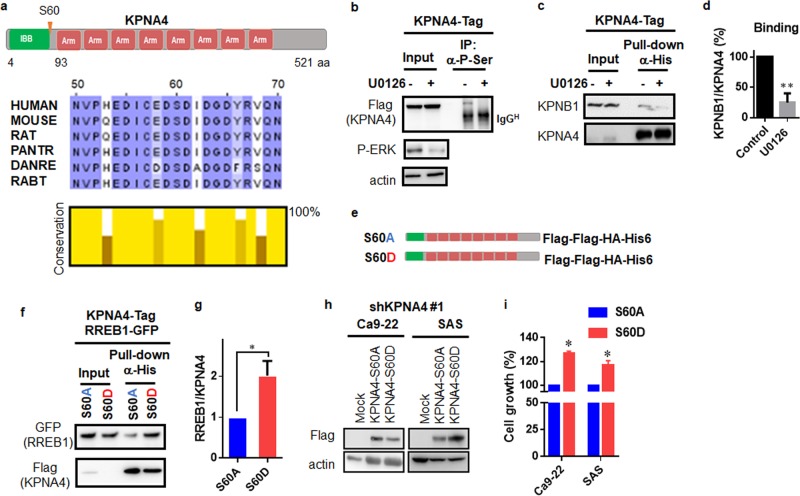
Phosphorylation of KPNA4 at S60 determines KPNA4 transport activity. **a** Schematic representation of the structural and functional domains and phosphorylation site of KPNA4 (upper panel), and evolutionarily conserved IBB domain in KPNA4 protein (bottom panel). **b**–**d** SAS stably expressing KPNA4-tag was subjected to IP analysis under MAPK inhibition. At 24 h treatment of U0126 (10μM), cells were harvested, lysed, and proceeded for pull-down assay using antiphospho-Ser antibody (**b**) or His-tag pull-down assay (**c**). Quantification of the interaction between KPNB1 and KPNA4 (**d**). Relative interacting amounts was normalized by KPNA4 levels, then amounts of KPNB1 without U0126 was considered as 100% (*n* = 3). One sample *t* test was performed using GraphPad QuickCalcs. **p < 0.01. **e** Diagram of mutation conducted in KPNA4. **f** Interactions of KPNA4 and RREB1 were addressed by western blot analysis. At 48 h after transfection, cells were harvested, lysed, and proceeded for His-tag pull-down assay. **g** Quantification of the interaction between KPNA4 and RREB1. Relative interacting amounts were normalized by KPNA4 levels, then amounts of RREB1 interacting KPNA4S60A was considered as 100% (*n* = 3). One sample *t* test was performed using GraphPad QuickCalcs. *p < 0.05. **h** Western blot analysis of exogenous KPNA4S60A or KPNA4S60D. **i** Short-term proliferat**i**on ability was analyzed MTT assay using HNSCCs expressing either KPNA4^S60A^ or KPNA4^S60D^ under shKPNA4 expression. Data represent means ± SD (*n* = 3). One sample *t* test was performed using GraphPad QuickCalcs. *p < 0.05.

**Fig. 6:**
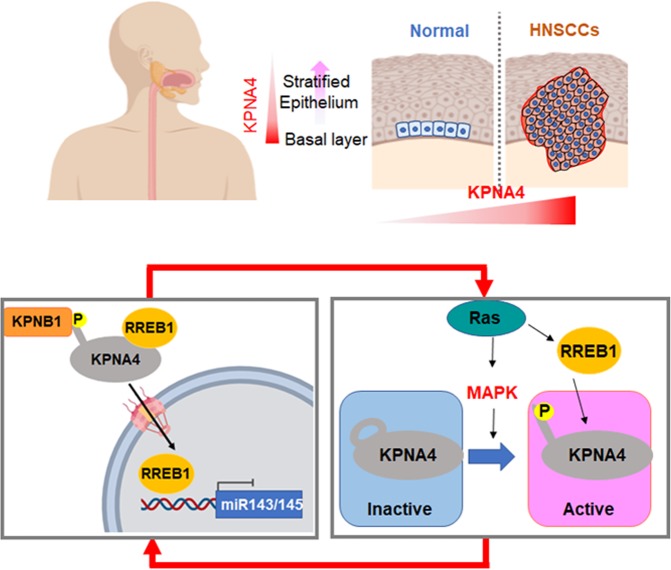
Hypothetical model of KPNA4 action in regulating Ras/MAPK signaling.

A previous study showed that Ser60 of KPNA4, in a well-conserved region (Fig. 5a), is phosphorylated in EGF stimulation-dependent manner [29]. Our results showed that KPNA4 establishes Ras-MAPK signaling. Therefore, we further examined the possible functions of phosphorylation of KPNA4 at Ser60 in Ras-MAPK signaling. Consistent with previous findings [29], our IP analysis demonstrated that phosphorylation levels (Ser) of KPNA4 strikingly decreased after MAPK inhibition (Fig. 5b). Moreover, blockage of phosphorylation of KPNA4 attenuated KPNA4/KPNB1 complex formation (Fig. 5c, d). We next generated an alanine- and asparatic acid-substituted mutant, KPNA4^S60A^ and KPNA4^S60D^, respectively (Fig. 5e), and performed IP analysis to examine the interaction between KPNA4 and RREB1 (Fig. 5f, g). KPNA4^S60D^ showed enhanced KPNA4/RREB1 complex formation compared with KPNA4^S60A^ (Fig. 5f, g). Furthermore, HNSCC cells expressing KPNA4^S60D^ grew significantly more than those with KPNA4^S60A^ (Fig. 5h, i). Together, this indicates that MAPK signaling augments KPNA4/cargo interaction though phosphorylation of Ser60 of KPNA4 (Fig. 6).

## Discussion

Here we identified KPNA4 as a specific KPNA subtype that shows genomic amplification and overexpression in HNSCC. The expression of KPNA4 is required for cellular proliferation and migration as well as preventing epidermal differentiation in HNSCC cells. We also demonstrated that KPNA4 mediates RREB1 transport and establishes a feed-forward cascade that potentiates Ras/ERK signaling in HNSCC.

Genomic DNA copy number aberration is frequent in cancer and can contribute to cancer initiation, growth, and survival. Our genomic analysis in human HNSCC showed that *KPNA4* was specifically amplified in HNSCC among the *KPNA* family. Consistent with the genomic profiles, overexpression of KPNA4 at mRNA levels was also specifically observed in HNSCC. The loss of function assay revealed that KPNA4-mediated nuclear transport is required for HNSCC proliferation. Recent studies demonstrated that certain karyopherins are elevated in several cancers and can regulate malignant phenotypes by affecting cytoplasm-nuclear transport systems [25, 30–34]. Taken together, these results indicate that targeting disease-specifically altered transport systems may serve as promising therapeutic strategies for cancer treatment.

Very recently, Yang et al., reported that an abnormal expression of KPNB1 lead to enhance c-MYC nuclear transport in prostate cancer, which establish feed-forward loop to maintain transcriptional KPNB1 expressions [25]. Although elevated amounts of KPNB1 in HNSCC were detected (Supplementary Fig. 5), the expression levels of KPNA2 rather than KPNA4 strongly related with activation of c-MYC targets in HNSCC (Supplementary Fig. 6a–e). Importantly, the expression amounts of KPNA2 was comparable between HNSCC and prostate cancers, whereas KPNA4 is most abundant in HNSCC (Fig. 1e, Supplemental Fig. 6f). Since overexpression of c-MYC is common in SCCs, it would be interesting whether the KPNB1/KPNA2 axis regulates c-MYC nuclear transport.

Tumor differentiation is an important clinical–pathological factor that affects the malignant potential of HNSCC. Here, we found that KPNA4 expression levels determine epidermal differentiation. Although epidermal differentiation is mainly regulated by transcriptional control of gene regulatory networks, we found that the expression level of KPNA4 is also crucial for epidermal differentiation in HNSCC cells. Previous studies demonstrated that mouse embryonic stem cells require the switching of KPNA subtypes during neural differentiation [22]. Collectively, these data strongly suggest that the HNSCC-specifically altered KPNA4 is a functional requisite for HNSCC biology as well as cell fate determination.

RREB1 is an oncogenic TF that suppresses miR143/145 expression to establish Ras/ERK oncogenic signaling in several cancers [27, 28]. However, the regulation of RREB1 nuclear transport has been unknown. We found that nuclear transport of RREB1 is mediated by the KPNA4/KPNB1 transport system that targets an NLS on RREB1. In addition, upon KPNA4 depletion, miR143/145 targeted not only Ras but also RREB1 in some cells (Supplementary Fig. 7) [27], therefore blockage KPNA4-RREB1 transport has advantage to effectively suppress Ras/ERK signaling in HNSCC.

Previous studies showed that KPNA4 was the sole transporter for NF-κB [35], and Yang et al. recently demonstrated KPNA4 mediates cancer metastasis through NF-κB transport in prostate cancer [32]. In addition, enhanced metastatic state of esophagus SCC by activation of KPNA4-mediated NF-kB pathway is reported very recently [36]. Consistent with previous reports, we also found that NF-κB was highlighted as a potent cargo of KPNA4 in both CARRIE and pathway analysis. We also found that the NF-κB inhibitor suppressed the growth of HNSCC cells (Supplementary Fig. 8). An involvement of NF-κB in SCC initiation and progression in vivo was previously demonstrated [37, 38]. Since KPNA4 functions as a transporter for these two oncoproteins, how these TFs coordinate in the evolution of SCC should be examined in future studies.

Our findings demonstrated that MAPK signaling enhanced the trafficking activity of KPNA4 via phosphorylation of Ser60 of KPNA4. KPNAs generally recognize the NLS in the cargo through its heat domain [19]. As the IBB domain, also the KPNB1-binding domain, of KPNAs can function as an NLS, the IBB is essential for the auto-inhibitory function of KPNAs through self-interaction between the internal IBB and heat domain [39]. Previous studies revealed that phosphorylation of cargo near the NLS can prevent the nuclear import of cargo [40, 41], supporting our conclusion that phosphorylation of Ser60 of KPNA4 determines the KPNA activity.

In summary, our results demonstrated that KPNA4, which is specifically upregulated in HNSCC, prevents epidermal differentiation and establishes feed-forward oncogenic signaling through RREB1 nuclear transport in HNSCC. In light of the importance of oncogenic TF transport regulation established by overexpression and posttranslational modification of KPNA4, targeting the systematic nuclear transport process mediated by KPNA4 represents a promising therapeutic approach for HNSCC.

## Materials and methods

### Cell culture

HEK293T cells, nontransformed human keratinocyte HaCaT, and HNSCC cell lines (HSC2, HSC3, Ca9-22 and SAS) were obtained from ATCC and maintained in Dulbecco’s Modified Eagle Medium (DMEM) supplemented with 10% (vol/vol) fetal bovine serum and 1% (vol/vol) penicillin/streptomycin (P/S) at 37 °C, 5% CO_2_ in a humidified atmosphere.

### Cell proliferation assay

HNSCC cells were seeded into a 96-well plate at 3000 cells/well and cultured for the indicated times. Cell viability was assessed using the MTT (3-(4, 5-dimethylthiazol-2-yl) -2, 5-diphenyltetrazolium bromide) method. In brief, 10 μL of 12 mM MTT solution was added into each well followed by 3 h incubation. The reaction was stopped by adding 100 μL of STOP solution (2% acetic acid, 16% SDS, 42% DMF). Samples were mixed thoroughly, and absorbance was measured at 570 nm.

### Foci formation assay

HNSCC cells were seeded into six-well plates at 1000 cells/well and cultured for 10 days. Cells were then fixed, stained with crystal violet and imaged using LAS4000 (Fujifilm, Aichi, Japan). The number of colonies (>0.1 mm^2^) was determined using Multi Gauge Ver3.0 (Fujifilm).

### Western blotting

Cells were lysed with lysis buffer (20 mM HEPES (pH 7.4), 350 mM sodium chloride, 1.5 mM magnesium chloride, 1 mM EGTA, 10% (v/v) glycerol, 1% Triton X-100, a mixture of protease inhibitors (Roche), 0.2 mM sodium orthovanadate, and 1 mM phenylmethylsulfonyl fluoride). Regarding samples subjected to nuclear transport analysis, nuclear-cytoplasmic fractionation was performed by following the manufacture’s protocol (NE-PER^TM^ Nuclear and Cytoplasmic Extraction reagents, ThermoFisher). Samples were subjected to SDS-PAGE followed by conventional wet transfer. Membranes were incubated with primary antibodies and exposed to secondary horseradish peroxidase-conjugated antibodies (Millipore). Images were detected by using an LAS-4000 image analyzer (Fujifilm). All antibodies used in this study are listed in Supplementary information, Table S1.

### Immunoprecipitation

Stably KPNA4 expressing HNSCC cells or HEK293T cells were transiently transfected with the indicated mammalian expression plasmids and harvested 72 h after transfection. Antibody was added and samples were incubated overnight at 4 °C. Samples were washed three times with lysis buffer and Dynabeads Protein A/G (VERITUS, Tokyo, Japan) were added. After 2 h, beads were washed four times in IP buffer (50 mM Tris, pH 7.6, 100 mM NaCl, 2 mM EDTA, 0.2% Nonidet P-40) and then subjected to western blot. For His-tag pull-down assay, all steps were performed following the manufacturer’s protocol (Dynabeads^TM^, His-Tag Isolation & Pulldown, 10103D, Invitrogen).

### Tissue slide and immunofluorescent analysis

A human skin tissue block (118–2795) was obtained from US Biomax Inc. Slides were deparaffinized and blocked with goat serum for 30 min at room temperature, followed by incubation with mouse anti-KPNA4 (PA5-18239, Thermo Scientific) and rabbit anti-IVL (1:100; SAB4501594, Sigma) overnight at 4 °C. After three washes, slides were incubated with Alexa 488 and Alexa 568 linked secondary antibody (Life Technologies) for 60 min. After three washes, Pro-Long Gold Antifade reagent (Life Technologies) was mounted onto samples and examined by confocal microscopy (objective × 60/1.2, FluoView^®^ FV10i, Olympus).

### Microscopic analysis

Cells on coverslips were incubated under indicated conditions. Cells were fixed for 10 min in 4% paraformaldehyde in PBS and then permeabilized with 0.3% Triton X-100 in PBS for 3 min at room temperature. Coverslips were incubated with indicated primary antibody for 2 h. Coverslips were washed three times and incubated with Alexa Fluor-conjugated secondary antibody (Life Technologies) for 1 h. After three washes, samples were mounted onto coverslips using Pro-Long Gold Antifade reagent (Life Technologies) and examined by confocal microscopy (objective × 60/1.2, FluoView^®^ FV10i, Olympus).

### cDNA vectors and siRNAs

Constructs for overexpressing KPNA4 were generated by cloning cDNA derived from HaCaT cells into the pLEX vector (Open Biosystems), C-terminal FLAG-HA-His6 tag, at NotI/AgeI sites. The KPNA4 coding sequences harboring mutations (S60A or S60D) were generated with the following primers: S60A-F: 5′-GAAGACGCTGATATAGATGGTGATTAT-3′; S60A-R: 5′- TATATCAGCGTCTTCACAGATATCTTC-3′; S60D-F: 5′-GAAGACGATGATATAGATGGTGATTAT-3′; and S60D-R: 5′-TATATCATCGTCTTCACAGATATCTTC-3′. Constructs for overexpressing GFP-fused RREB1 were produced by cloning the ORF derived from pSPORT-RREB1 (#41145, Addgene) into pN1-EGFP using XhoI/AgeI sites. Scramble siRNA (D-001210-01) was purchased from Thermo Scientific.

### Transfections, viral particle production, and infection

DNA and siRNA transfections were performed using Lipofectamine 2000 and Lipofectamine RNAiMAX (Life Technologies), respectively. Lentiviral particles were produced with the MISSION Lentiviral Packaging System (Sigma-Aldrich). HNSCC cells were transduced with the lentiviral particles in the presence of 8 μg/ml polybrene (Sigma-Aldrich) for 48 h according to a previous report [42].

### cDNA preparation and quantitative real-time RT-PCR assay

We used 500 ng RNA for cDNA preparation using ReverTra Ace^®^ qPCR RT Master Mix (TOYOBO). Quantitative real-time RT-PCR was performed by SYBR^®^ Premix Ex Taq™ II (Takara) in a Thermal Cycler Dice^®^ Real Time System (Takara) according to the manufacturer’s instructions. The relative mRNA expression level of target genes was calculated using GAPDH mRNA as a loading control. Primer sequences are listed in Supplementary information, Table S2. For miRNA analysis, small RNA was prepared by following the manufacturer’s protocol (Wako, ISOGEN, 311-02501). Reverse transcription and qPCR was performed using the Luna® Universal One-Step RT-qPCR Kit. Primers (Supplementary information, Table S2) were designed according to a previous report [43].

### DNA microarray analysis

Cyanine-3-labeled cRNA was prepared from 0.2 μg RNA using the Low Input Quick Amp Labeling Kit (Agilent Technologies) according to the manufacturer’s instructions, followed by RNeasy column purification (QIAGEN, Valencia, CA). Samples were hybridized to a Whole Human Genome microarray 4 × 44K Ver. 2.0 (G4845A, Agilent Technologies). The slides were scanned on the Agilent DNA Microarray Scanner (G2539A) using one color scan setting for 4 × 44k array slides. The scanned images were analyzed with Feature Extraction Software 11.0.1.1 (Agilent) using default parameters (protocol AgilentHD_GX_1Color and Grid: 026652_D_F_20120130) to obtain background subtracted and spatially detrended Processed Signal intensities. The raw data has been deposited in the Gene Expression Omni-bus (GEO) database, under number GSE128853.

### Bioinformatics and data analysis

Genomic amplification status and mRNA expression levels of KPNA family in HNSCCs were obtained from TCGA. GO analysis and pathway were performed via ConsensusPathDB (see “URLs”). GSEA was performed with GSEA v2.2.2 software (see “URLs”).

### Statistical analyses

Cell proliferation assay, anchorage-dependent colony formation assay, and real-time RT-PCR analysis were performed in triplicate (*n* = 3) and independently replicated three times. Data are the average of experimental replicates (*n* = 3). Unpaired two-tailed *t* test was performed by GraphPad Prism in the statistical analysis of cell proliferation assay, anchorage-dependent colony formation assay, and real-time RT-PCR analysis. *P* < 0.05 was considered statistically significant. When control group is considered as 100%, one sample *t* test was performed using GraphPad QuickCalcs (see “URLs”).

### URLs

TCGA, http://www.broadinstitute.org/igv; cBio Cancer Genomics Portal, http://www.cbioportal.org/; GEO, Cancer Cell Line Encyclopedia (CCLE), http://portals.broadinstitute.org/ccle/; GSEA, http://software.broadinstitute.org/gsea; ConsensusPathDB, http://consensuspathdb.org/; GraphPad QuickCalcs; http://graphpad.com/quickcalcs/OneSampleT1.cfm. CARRIE, https://zlab.bu.edu/CarrieServer/html/.

## Supplementary information

Supplementary information

Figure S1

Figure S2

Figure S3

Figure S4

Figure S5

Figure S6

Figure S7

Figure S8

Table S1 and Table S2
