# Supplementary material for: Disease-specific alteration of karyopherin-α subtype establishes feed-forward oncogenic signaling in head and neck squamous cell carcinoma
Source: Oncogene. 2019 Dec 10;39(10):2212–23. doi: 10.1038/s41388-019-1137-3 (PMC7056645; doi:10.1038/s41388-019-1137-3)
Supplement: Supplementary file 1 — Supplementary information [file 41388_2019_1137_MOESM1_ESM.docx]

**Supplementary information**

**Inhibitors and siRNA**

Importazol (IPZ) was purchased from SIGMA and prepared at 10 mM stock. JSH-23 (NF-κB inhibitor) was obtained from Millipore, and TNF-α was from (Wako). siRNA for KPNB1 is from SIGMA.

**Generation of GFP-NLS**

GFP with SV40-derived NLS (PKKKRKV) was generated by inverse PCR approach using following primers; F: 5’-CGCAAGGTATAAAGCGGCCGCGACTCTAGATCATAATCA-3’, Reverse: 5’-CTTTTTCTTGGGCTTGTACAGCTCGTCCATGCCGAGAGTGAT-3’

**Microscopic analysis**.

293T cells on coverslips were transitory expressed either GFP or GFP^NLS^. Cells were fixed for 10 min in 4% paraformaldehyde in PBS and then permeabilized with 0.3% Triton X-100 in PBS for 3 min at room temperature. After three washes, samples were mounted onto coverslips using Pro-Long Gold Antifade reagent (Life Technologies) and examined by confocal microscopy (objective × 60/1.2, FluoView® FV10i, Olympus).

**Migration assay**

HNSCC cells were seeded onto transwell inserts (3422, Corning, Kennebunk, ME, USA) in 24-well plates and incubated for 48 h. The inserts were washed with PBS, and non-migrating cells were wiped off the top side. Migrated cells were fixed with 4% paraformaldehyde, stained with crystal violet, and cells were counted.

**In vitro irradiation**

Radiation exposures (150 kVp, 20 mA, 0.5-mm Al and 0.3-mm Cu filters) were performed using an X-ray generator (MBR-1520R-3, Hitachi Medical Corporation, Tokyo, Japan) at a distance of 45 cm from the focus at a dose rate of 1.00-1.02 Gy/min.

**Cell cycle analysis**

HNSCC cells (SAS) expressing either shRNA for non-targeting or KPNA4 were harvested and fixed in 70% ethanol overnight at −20°C. The fixed cells were washed with Ca^(2+)^- and Mg^(2+)^- free phosphate-buffered saline [PBS(−)] and then treated with RNase A (200 µg/mL) at 37°C for 30 min to hydrolyze RNA. After treatment, the cells were washed with PBS(−) and stained with PI (30 µg/mL) for 30 min in the dark. Then, the cells were filtered with cell strainer (BD Falcon^TM^, Franklin Lakes, NJ, USA). The cell-cycle distribution was analyzed by flow cytometry (Cytomics FC500, Beckman–Coulter).

**Analysis of apoptosis**

Cell death was analyzed by annexin V-FITC (BioLegend) and PI staining according to the manufacturer’s instructions. In brief, the cells treated with each compound were harvested, washed, and suspended in annexin V Binding Buffer (BioLegend). The annexin V-FITC (2.5 µg/mL) and PI solution (50 µg/mL) were added to the cell suspension and incubated for 15 min at room temperature in the dark. Then, the apoptotic cells were analyzed by flow cytometry (Cytomics FC500, Beckman–Coulter). In the annexin V/PI quadrant gating, annexin V(−)/PI(−), annexin V(+)/PI(−), and annexin V(+)/PI(+) were used to identify the fraction of viable cells, early apoptotic cells, and late apoptotic/necrotic cells, respectively.

**Cell proliferation assay.** HNSCC cells silenced KPNB1 were seeded into a 96-well plate at 3,000 cells/well and cultured for the indicated times. In the IPZ or JSH-23 treatment, culture medium contained those inhibitors at each concentration during assay. Cell viability was assessed using the MTT (3-(4, 5-dimethylthiazol-2-yl) -2, 5-diphenyltetrazolium bromide) method. In brief, 10 μL of 12 mM MTT solution was added into each well followed by 3 h incubation. The reaction was stopped by adding 100 μL of STOP solution (2% acetic acid, 16% SDS, 42% DMF). Samples were mixed thoroughly, and absorbance was measured at 570 nm.

**Supplementary figure legends**

**Supplemental Fig. 1.** The effects of SV40-derived NLS (PKKKRKV) on nuclear localization. **a** Schematic representation of the structure of artificial GFP fused with SV40 derived NLS-seq. **b** Representative image of both GFP and GFPNL in HEK293T cells.

**Supplementary Fig. 2.** KPNA4 silencing inhibited malignant phenotypes in HNSCCs. **a, b** Long-term growth ability (a) and migration activity (b) were analyzed in HNSCC cells upon KPNA4 knock-down. **c, d** Radiation-induced apoptosis was determined by flow-cytometric analysis by sub-G1 fraction (c) or annexin-V/PI co-staining (d). Radiation exposures (150 kVp, 20 mA, 0.5-mm Al and 0.3-mm Cu filters) were performed using an X-ray generator (MBR-1520R-3, Hitachi Medical Corporation, Tokyo, Japan) at a distance of 45 cm from the focus at a dose rate of 1.00-1.02 Gy/min. Data show mean ± SD from three independent experiments (*n*=3). One sample t-test or Student’s t-test or Mann-Whitney *U-Test* was employed according to data distributions (*:*p*<0.05. **:*p*<0.01).

**Supplementary Fig. 3.** Blockage of KPNB functions suppresses proliferation in SCC**. a** Short-term proliferation was analyzed by MTT assay. Importazol (IPZ) was obtained from Wako. **b** Western blot analysis of KPNB1. **c** Short-term proliferation of KPNB1-silenced cells was analyzed by MTT assay. Data show mean ± SD from three independent experiments (*n*=3). *P*- values are based on *Student t-test* (*:*p*<0.05. **:*p*<0.01).

**Supplementary Fig. 4.** RREB1 mediates MAPK activation through suppressing miR-143/145 levels in SCC cells. **a** qRT-PCR analysis of miR-143 and miR-145 in HNSCC cell lines depleted of KPNA4. Small RNA was prepared by following protocol (Wako, ISOGEN, 311-02501). Revers-transcription and qPCR was performed using (Luna® Universal One-Step RT-qPCR Kit Protocol). Expression levels of cells expressing non-targeting shRNA is considered 100%. Data show mean ± SD from three independent experiments (*n*=3). P values are based on one sample t-test with *indicating p<0.05 and **p<0.01. **b** Western blot analysis of activated forms of ERK. **c** Short-term proliferation was analyzed by MTT assay. Data show mean ± SD from three independent experiments (*n*=3). P values are based on *Student t-test* with *indicating p<0.05 and **p<0.01. **d** RREB1 expression profiles from GTEX-portal.

**Supplementary Fig. 5**. Expression profiles of KPNB1 in HNSCC. **a** mRNA levels of KPNB1 in normal and tumor samples extracted through Cancer RNA-seq Nexus (CRN, http://syslab4.nchu.edu.tw/index.jsp). **b** Comparison of mRNA levels between normal and tumor samples from each different stage. **c** Co-expression analysis of mRNA for KPNB1 and KPNA4 through cBioportal (n=515). **d** High KPNB1 expression (mRNA expression z-Scores (RNA Seq V2 RSEM)>mean + 2.0 SD) was associated with poor overall survival of HNSCC patients in the TCGA cohorts.

**Supplementary Fig. 6.** Correlation among MYC-targets and KPNA subtypes in HNSCC.

**a** Co-expression analysis of mRNA for MYC-targets and KPNA family through cBioportal (n=515). **b** Pearson r value (CCNB1 or CDK1 vs karyopherin family) obtained from cBioportal. **c** Co-expression analysis of mRNA for KPNB1 and KPNA subtypes through cBioportal (n=515). **d** Pearson r value (KPNB1 vs KPNA subtypes) obtained from cBioportal. **e** qRT-PCR analysis of CCNB1 (CyclinB1) and CDK1 mRNA levels upon KPNA4 knock-down. Data show mean ± SD from three independent experiments (*n*=3). **f** KPNA2 mRNA expression across different types of cancer cells from CCLE. One-way ANOVA was performed.

**Supplementary Fig. 7.** The effects of KPNA4 depletion on the transcript amounts of RREB1 and NRAS. qRT-PCR analysis of RREB1 and NRAS in HNSCC cell lines upon KPNA4 knock-down. xpression levels of cells expressing non-targeting shRNA is considered 100%. Data show mean ± SD from three independent experiments (n=3). P values are based on one sample t-test (*:*p*<0.05. **:*p*<0.01).

**Supplementary Fig. 8.** Inhibition of NF-κB pathway suppresses cellular growth of HNSCCs. **a** Western blot analysis of p65 using fractionated samples from SCC cells following indicated treatments. TNF-α stimulation was used to enhance p65 nuclear translocation. **b** Short-term proliferation was analyzed by MTT assay. Data show mean ± SD from three independent experiments (n=3). P values are based on one sample *t-test.*
