# Supplementary figures and images for: Disease-specific alteration of karyopherin-α subtype establishes feed-forward oncogenic signaling in head and neck squamous cell carcinoma
Source: Oncogene. 2019 Dec 10;39(10):2212–23. doi: 10.1038/s41388-019-1137-3 (PMC7056645; doi:10.1038/s41388-019-1137-3)

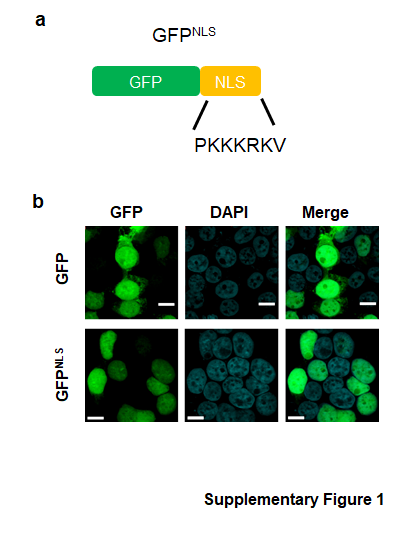

Supplement: Supplementary file 2 — Figure S1 [file 41388_2019_1137_MOESM2_ESM.tif]

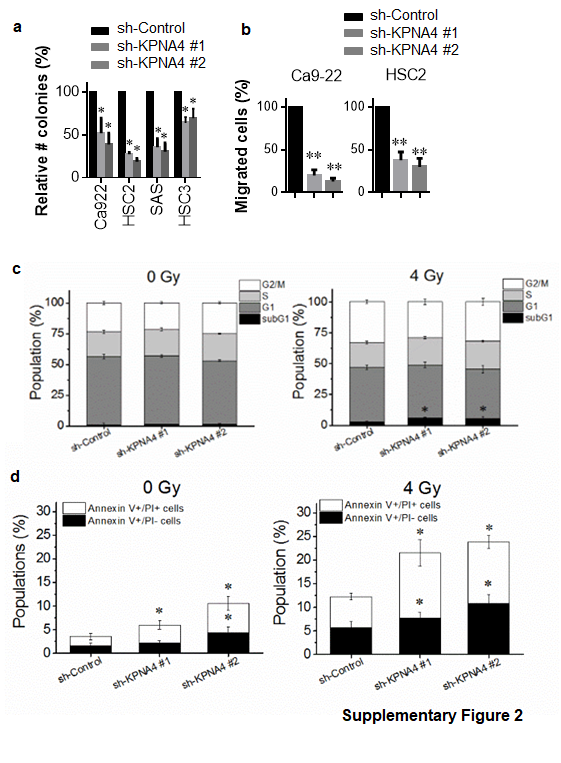

Supplement: Supplementary file 3 — Figure S2 [file 41388_2019_1137_MOESM3_ESM.tif]

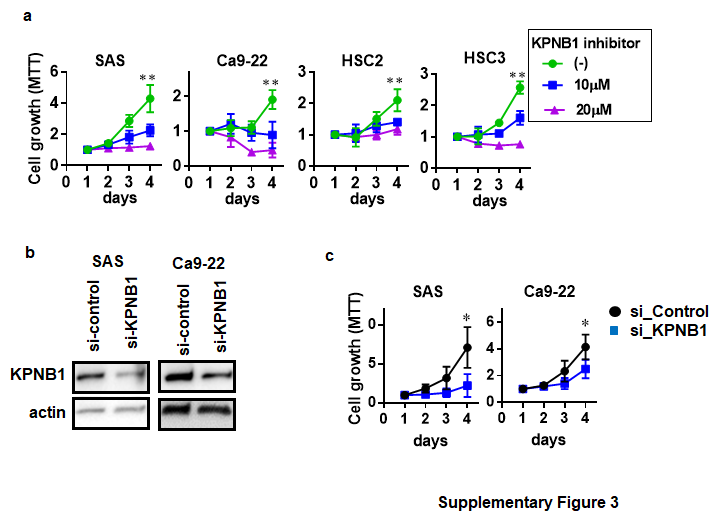

Supplement: Supplementary file 4 — Figure S3 [file 41388_2019_1137_MOESM4_ESM.tif]

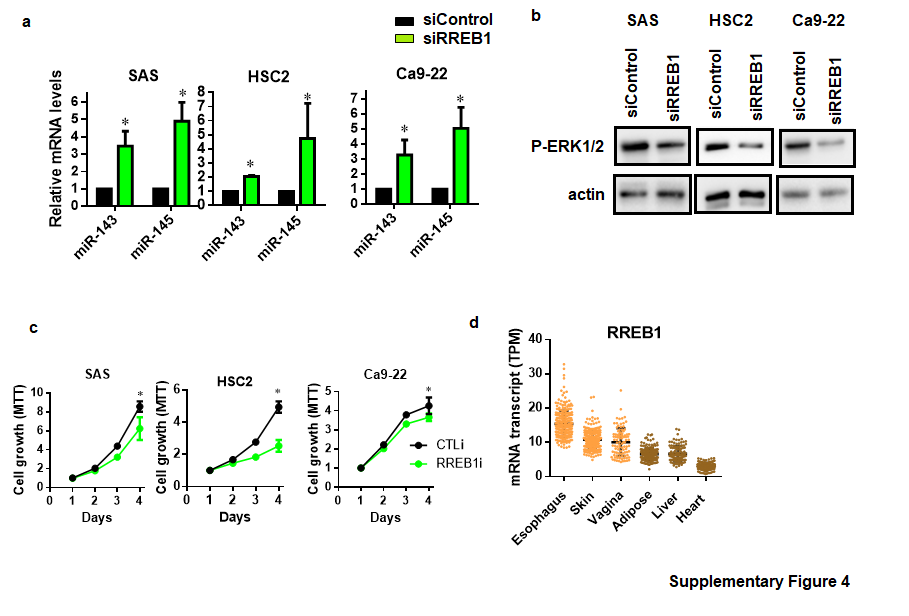

Supplement: Supplementary file 5 — Figure S4 [file 41388_2019_1137_MOESM5_ESM.tif]

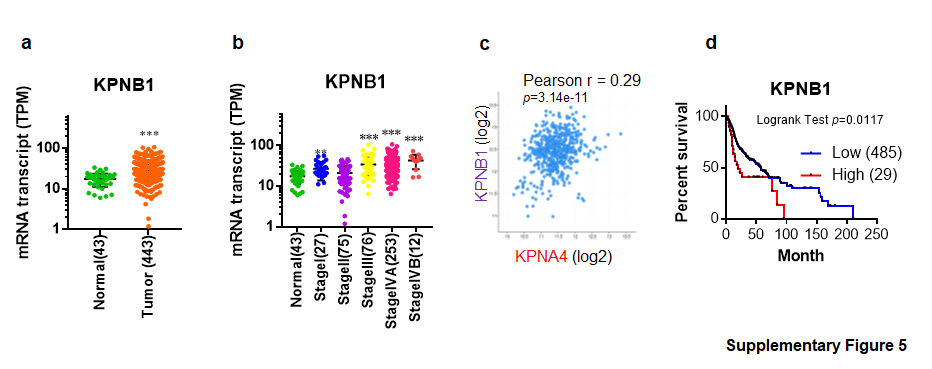

Supplement: Supplementary file 6 — Figure S5 [file 41388_2019_1137_MOESM6_ESM.tif]

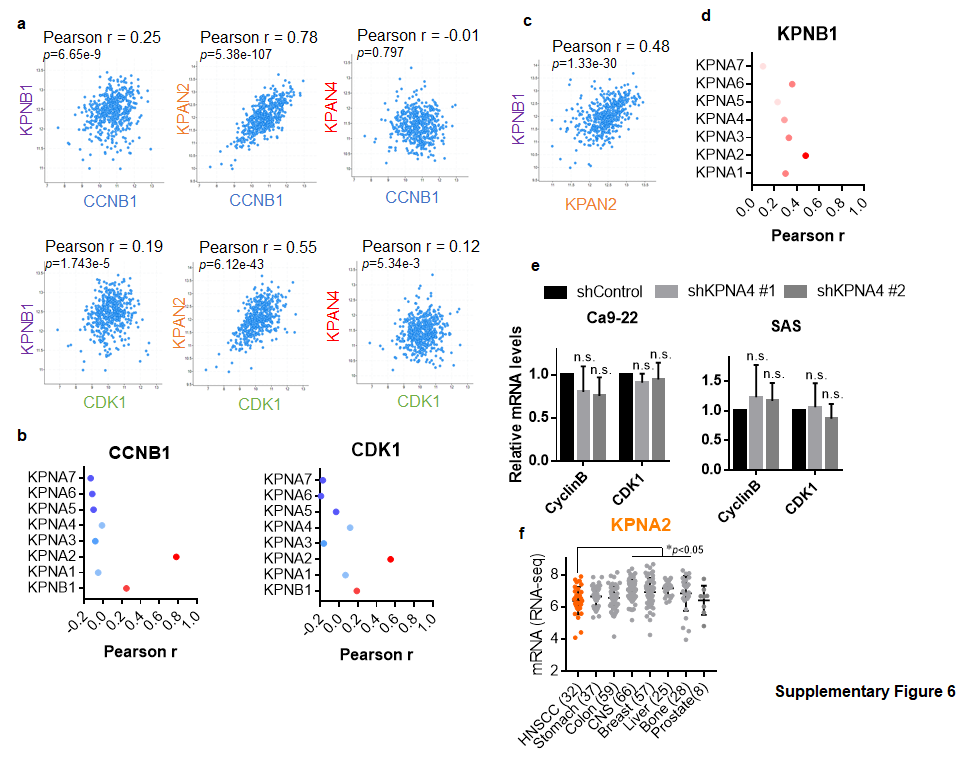

Supplement: Supplementary file 7 — Figure S6 [file 41388_2019_1137_MOESM7_ESM.tif]

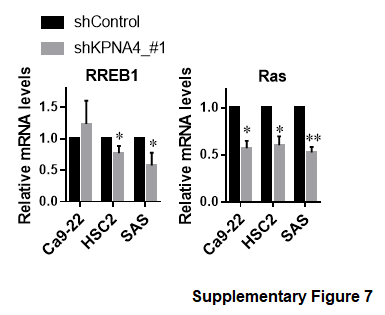

Supplement: Supplementary file 8 — Figure S7 [file 41388_2019_1137_MOESM8_ESM.tif]

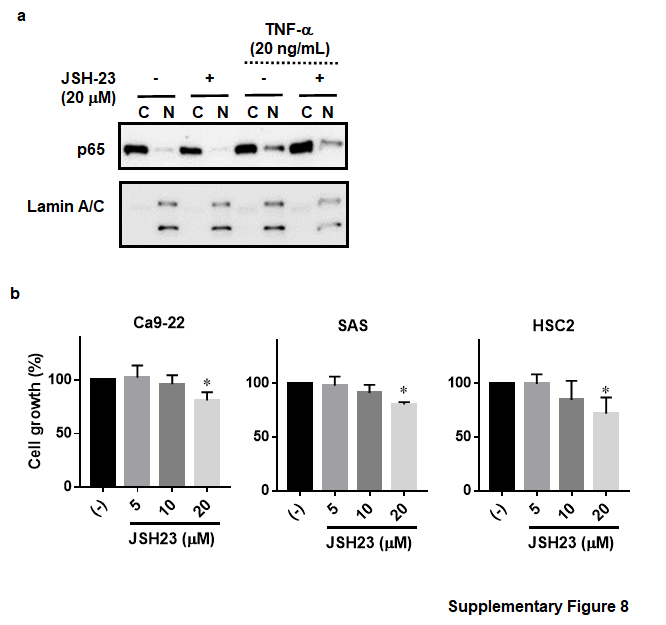

Supplement: Supplementary file 9 — Figure S8 [file 41388_2019_1137_MOESM9_ESM.tif]
