# Supplementary material for: Disease-specific alteration of karyopherin-α subtype establishes feed-forward oncogenic signaling in head and neck squamous cell carcinoma
Source: Oncogene. 2019 Dec 10;39(10):2212–23. doi: 10.1038/s41388-019-1137-3 (PMC7056645; doi:10.1038/s41388-019-1137-3)
Supplement: Supplementary file 10 — Table S1 and Table S2 [file 41388_2019_1137_MOESM10_ESM.docx]

| Table1. Antibodies |  |  |
| --- | --- | --- |
|  |  |  |
| Target | Catalog # | Company |
| anti-hImportin α3 (KPNA4) | MAB8204 | R&D system |
| β-Actin | sc-47778 | Santa Cruz |
| Karyopherin β1 (KPNB1) | sc-137016 | Santa Cruz |
| Anti DYKDDDDK tag | 040-30953 | Wako |
| Anti GFP | 012-20461 | Wako |
| RREB1 | HPA001756 | Sigma |
| p42/44 MAPK (Erk 1/2) | #9102 | Cell Signaling Technology |
| Phospho-p44/42 MAPK (Erk1/2) (Thr202/Tyr204) | #9101 | Cell Signaling Technology |

| Table 2. primers |  |
| --- | --- |
|  | shRNA Oligo-seq (5 to 3) |
| shKPNA4_#1-F | CCGGGCGGAACATTTGGTTTCAATTCTCGAGAATTGAAACCAAATGTTCCGCTTTTTG |
| shKPNA4_#1-R | AATTCAAAAAGCGGAACATTTGGTTTCAATTCTCGAGAATTGAAACCAAATGTTCCGC |
| shKPNA4_#2-F | CCGGGCACAAGTTGTGCAAGTAGTACTCGAGTACTACTTGCACAACTTGTGCTTTTTG |
| shKPNA4_#2-R | AATTCAAAAAGCACAAGTTGTGCAAGTAGTACTCGAGTACTACTTGCACAACTTGTGC |
| Primers for RT-PCR |  |
| GAPDH-F | GAAGGTGAAGGTCGGAGTC |
| GAPDH-R | GAAGATGGTGATGGGATTTC |
| RREB1-F | ATAACTGCCCCCTGTGTGAG |
| RREB1-R | TGTCTGTGCATGTTCCCATT |
| NRAS-F | AAACCTCAGCCAAGACCAGA |
| NRAS-R | CCCTGAGTCCCATCATCACT |
| IVL-F | TTACTGTGAGTCTGGTTGAC |
| IVL-R | TGTTTCATTTGCTCCTGATG |
| S100P-F | AAGGTGCTGATGGAGAAGGA |
| S100P-R | ACTTGTGACAGGCAGACGTG |
| SPRR1A-F | GCCACTGGATACTGAACA |
| SPRR1A-R | AGGAAGACTAGGGATGGTT |
| Primers for miRNA |  |
| Universal reverse primer | GTGCAGGGTCCGAGGT |
| stem-loop-143 | GTTGGCTCTGGTGCAGGGTCCGAGGTATTCGCACCAGAGCCAAC ACCAGA |
| stem-loop-145 | GTTGGCTCTGGTGCAGGGTCCGAGGTATTCGCACCAGAGCCAAC AGGGAT |
| stem-loop-SNOR | GTTGGCTCTGGTGCAGGGTCCGAGGTATTCGCACCAGAGCCAAC AGTCAG |
